# Iraqi health system in kurdistan region: medical professionals' perspectives on challenges and priorities for improvement

**DOI:** 10.1186/1752-1505-4-19

**Published:** 2010-11-30

**Authors:** Nazar P Shabila, Namir G Al-Tawil, Rebaz Tahir, Falah H Shwani, Abubakir M Saleh, Tariq S Al-Hadithi

**Affiliations:** 1 Department of Community Medicine, College of Medicine, Hawler Medical University, Erbil, Iraq; 2 Directorate of Health, Erbil, Iraq

## Abstract

**Background:**

The views of medical professionals on efficiency of health system and needs for any changes are very critical and constitute a cornerstone for any health system improvement. This is particularly relevant to Iraqi Kurdistan case as the events of the last few decades have significantly devastated the national Iraqi health system while the necessity for adopting a new health care system is increasingly recognized since 2004. This study aims to examine the regional health system in Iraqi Kurdistan from medical professionals' perspectives and try to define its problems and priorities for improvement.

**Methods:**

A survey questionnaire was developed and administered to a convenience sample of 250 medical professionals in Erbil governorate. The questionnaire included four items; rating of the quality of services and availability of resources in the health institutions, view on different aspects of the health system, the perceived priority needs for health system improvement and gender and professional characteristics of the respondents.

**Results:**

The response rate to the survey was 83.6%. A high proportion of respondents rated the different aspects of services and resources in the health institutions as weak or very weak including the availability of the required quantity and quality of medicines (68.7%), the availability of sufficient medical equipment and investigation tools (68.7%), and the quality of offered services (65.3%). Around 72% of respondents had a rather negative view on the overall health system. The weak role of medical research, the weak role of professional associations in controlling the system and the inefficient health education were identified as important problems in the current health system (87.9%, 87.1% and 84.9%, respectively). The priority needs of health system improvement included adoption of social insurance for medical care of the poor (82%), enhancing the role of family medicine (77.2%), adopting health insurance system (76.1%) and periodic scientific evaluation of physicians and other health staff (69.8%).

**Conclusion:**

Medical professionals were generally unsatisfied with the different aspects of the health system in Iraqi Kurdistan region. A number of problems and different priority needs for health system improvement have been recognized that require to be studied in more details.

## Background

The major objective of a country's health system is to assure the health of the general public through offering good quality and prompt services according to the needs of the population [1]. The health system needs to go through a process of continuous changes and improvement in order to be able to cope with different changes in the health and population environments and to appropriately respond to different challenges and needs [2].

The history of formal health care system in Iraq began in early 1920s, but the Iraqi Ministry of Health (MoH) was established in 1952 and its organizational structure was formalized in 1959. This organizational structure has changed little since its establishment [3,4]. The health care system in Iraq adopts a hospital-oriented and capital-intensive model that requires large-scale imports of medicines and medical equipment [3,5]. In the 1970s and early 1980s, Iraq witnessed spectacular social and economic development leading to the development of an efficient health system that was considered one of the best in the Middle East region. This period was associated with improvements in several critical health outcomes [3,6,7]. However, the capacity and performance started to deteriorate during the 1980s and the 1990s as a result of two wars and economic sanctions leading to serious decline in indicators of population health outcome to levels comparable to some of the least developed countries [3,8].

With its establishment in early 1990s, the MoH of Iraqi Kurdistan Regional Government followed the basic organizational structure and system of the Iraqi MoH. In the public sector, the health services are provided through a network of primary health care (PHC) centers and hospitals where services are provided at very low charges to all people with equal chance for access. However, this has led to overuse of health services and overcrowding of health facilities with their adverse effects [3,5]. The significant devastation of the health system in Iraqi Kurdistan by the events of the last few decades together with latest demographic, political and economic evolutions have made the necessity for adopting a new health care system increasingly recognized [3].

Medical professionals have important role and power in adopting and running health-care systems. Therefore, their views on efficiency of such system and needs for any changes are very critical and constitute a cornerstone for any health system improvement [9,10]. While medical professionals' groups or associations have strongly influenced efforts in health care reform in many contexts, the collective views of individual medical professionals are often obscured. An extensive literature review has yielded only few studies that have directly examined the view of medical professionals in different aspects of health system reform [1,9,10].

Up to our knowledge only few studies have examined the medical professionals' perception of the health system in Iraqi Kurdistan region [11,12]. Given the enormity of the current effort to reform health system in Iraqi Kurdistan and its potential effect on future generations, policymakers need to hear the views of the whole range of medical professionals on the key elements of reform. Faced with this absence of empirical data, this paper aims to examine the health system in Iraqi Kurdistan region from medical professionals' perspectives and try to define its problems and priorities for improvement.

## Methods

This study was based on a self-administered questionnaire survey of medical professionals in Iraqi Kurdistan region. Iraqi Kurdistan is a self-ruling region, located in northern Iraq and comprised of three governorates out of the 18 governorates of Iraq; Erbil, Duhok and Sulaymaniya. Erbil governorate is the capital of the Iraqi Kurdistan region comprising eight administrative districts and inhabited by approximately two million persons [13]. There are 12 public hospitals, 197 primary health care (PHC) centers, 7 small private hospitals and a large number of private clinics in Erbil governorate [14] with around 1085 physicians, 250 dentists and 265 pharmacists working in the public health sector [4].

The study was carried out in 5 hospitals and 8 PHC centers located in Erbil city. The 5 hospitals included all public hospitals in Erbil city that were purposively selected as they contain a large number of medical professionals of different professional characteristics. Out of 14 main PHC centers in Erbil city, 8 were selected to be included in the study and these were purposively selected to represent sectors of different socioeconomic levels in Erbil city.

A convenience sample of 250 medical professionals (physicians, dentists and pharmacists) who have been engaged in direct patient care in the selected hospitals and PHC centers was selected. All the medical professionals available in these hospitals and PHC centers on the day of visit were selected. A convenience sampling strategy rather than a random one was adopted for this study since collection of all lists of medical professionals in Erbil governorate, random selection of participants and contacting them was logistically difficult especially that no funds were available for this study. A statistically representative sample size of medical professionals in Erbil governorate was established at 200 persons through choosing a 95% confidence in the result of an estimated 30% satisfaction with the health system and a representativity error of ± 6%. The sample size was increased to 250 in order to adjust for non-response.

A four item questionnaire was developed to assess medical professionals' rating of the quality of services and availability of resources in the health institutions, their view on different aspects of the health system, and finally identifying the priority needs for health system improvement. The questionnaire was developed based on a small open-end questionnaire survey involving 20 purposively selected medical professionals, extensive literature review, reviewing the World Health Organization (WHO) health system building blocks [15], and local experts' opinion. The survey instrument was tested and subjected to three cycles of modifications based on iterative feedback received from ten medical professionals in clinical practice.

The questionnaire was directly dispatched to the selected sample with a brief description of the purpose of the study, clarification of the type of questions and a request for an informed consent. Filled out questionnaires were collected on the next day. Follow-up visits were made for the participants who did not give back the completed questionnaire on the specified date. The survey was conducted between April 15 and July 15, 2009.

The respondents' view was assessed on different services provided and facilities available at their working institutions including the quality of offered health services, availability of the required quantity and quality of medicines, availability of medical equipments and tools and availability of sufficient number of nurses and other health care workers. The respondents were also asked to rate seven different aspects of the health system covering the whole health system, financing the health sector, staff salaries, role of private sector in comparison with public sector, health education activities, role of professional associations in controlling the health system or the private practice and role of medical research. The respondents were asked to rate their assessment on a five -point response scale from "very weak" to "very good".

The respondents' opinions about the priority needs for the improvement of the health system were assessed by asking them to rate on three-point response scale; "priority need", "some need" or "no need" of a list of 9 aspects of the health system. Even though some of the questions with the potentiality of being unclear were clarified to participants before handling them the questionnaire, the "do not know" response was also added for those who had no idea about any specific need for health system improvement. Data were also collected on respondents' gender, profession, place of work (hospital or health center), and current administrative position.

Statistical analysis involved only application of descriptive statistics. Responses "very weak" and "weak" were considered negative view, while the other responses were considered positive view. This study was approved by the Ethics Committee at Hawler Medical University.

## Results

Of 250 medical professionals who received the survey questionnaire, 209 individuals (83.6%) responded. There were no statistically significant differences between the gender and professional characteristics of respondents and non-respondents. The gender and professional characteristics of the respondents included; females: 40.7%; profession: physicians (63.6%), dentists (20.1%), pharmacists (16.3%); place of work: hospitals (86.6%), health centers (13.4%); personnel in management position: 18.2%. Out of 135 physicians participated in this study, 38.5% were interns, 15.6 were general practitioners, 28.9% were senior house officers or specialty trainees and 17.0% were specialist physicians.

A high proportion of respondents rated the different aspects of services and resources in their working institutions as weak or very weak including the offered services (65.3%), availability of the required quantity and quality of medicines (68.7%) and availability of sufficient medical equipment and investigation tools (68.7%). On the other hand, 64.3% of respondents were satisfied with the availability of sufficient number of nurses and other health care workers in their working institutions. Around 72% of respondents had a rather negative view on or dissatisfaction with the overall health system. More specific problems in the health system as identified by majority of respondents included the weak role of medical research (87.9%), the weak role of professional associations in controlling health system or private practice (87.1%) and inadequate health education activities (84.9%). Detailed respondents' rating of different services and resources in the working health institutions and different aspects of the health system are shown in Table 1.

**Table 1:** Respondents' rating of different services and resources in the working health institutions and different aspects of the health system

Variable	Scale [No. (%)]						
	
	Negative view			Positive view			
	
	Very weak	Weak	Total	Satisfactory	Good	Very good	Total
**Health institution aspects**							

Offered services	45 (21.7)	90 (43.5)	135 (65.2)	51 (24.6)	21 (10.1)	0 (0.0)	72 (34.8)

Availability of required quantity and quality of medicines	67 (32.2)	76 (36.5)	143 (68.8)	47 (22.6)	16 (7.7)	2 (1.0)	65 (31.3)

Medical equipment and investigation tools	61 (29.3)	82 (39.4)	143 (68.8)	47 (22.6)	17 (8.2)	1 (0.5)	65 (31.3)

Availability of sufficient number of nurse and other health care workers	25 (12.1)	49 (23.7)	74 (35.7)	67 (32.4)	50 (24.2)	16 (7.7)	133 (64.3)

**Health system aspects**							

Overall health system	50 (24.0)	100 (48.1)	150 (72.1)	47 (22.6)	11 (5.3)	0 (0.0)	58 (27.9)

Government fund allocation for health	38 (19.6)	92 (47.4)	130 (67.0)	43 (22.2)	20 (10.3)	1 (0.5)	64 (33.0)

Salary of medical professionals	25 (12.1)	64 (31.1)	89 (43.2)	66 (32.0)	48 (23.3)	3 (1.5)	117 (56.8)

Role of private sector compared with public sector	34 (17.2)	76(38.4)	110(55.6)	56 (28.3)	31 (15.7)	1 (0.5)	88 (44.4)

Health education activities	83 (40.5)	91 (44.4)	174 (84.9)	26 (12.7)	5 (2.4)	0 (0.0)	31 (15.1)

Role of professional associations in controlling health system and private practice	116 (57.4)	60 (29.7)	176 (87.1)	19 (9.4)	6 (3.0)	1 (0.5)	26 (12.9)

Role of medical research in health system	112 (54.4)	69 (33.5)	181 (87.9)	17 (8.3)	8 (3.9)	0 (0.0)	25 (12.1)

Note: Not all questions are answered by all respondents

The highest priority needs of health system improvement identified by respondents included social insurance for medical care of the poor (82%), enhancing the role of family medicine in the health system (77.2%), adopting health insurance system (76.1%), periodic scientific evaluation of physicians and staff (69.8%) and better role for the regional MoH and professional associations in controlling the private sector (61.5%). Details of the priority needs for health system improvement as identified by the respondents are shown in Table 2.

**Table 2:** Priority needs for health system improvement as identified by the respondents (n = 209)

Health system aspects	Priority need		Some need		No need		Don't know	
	
	No.	(%)	No.	(%)	No.	(%)	No.	(%)
Adopting health insurance system	153	(76.1)	35	(17.4)	5	(2.5)	8	(4.0)

Periodic scientific assessment of physicians and staff	143	(69.8)	46	(22.4)	13	(6.3)	3	(1.5)

Minimizing the gap between urban & rural health services (Equity)	117	(58.2)	72	(35.8)	7	(3.5)	5	(2.5)

Social insurance for medical care of the poor	168	(82.0)	32	(15.6)	3	(1.5)	2	(1.0)

Public-private systems separation	101	(50.5)	54	(27.0)	30	(15.0)	15	(7.5)

Better role for regional MOH and professional associations in controlling private sector	123	(61.5)	59	(29.5)	10	(5.0)	8	(4.0)

Privatization or self-financing of public hospitals	75	(37.5)	48	(24.0)	67	(33.5)	10	(5.0)

Privatization of PHC services	54	(26.5)	74	(36.3)	61	(29.9)	15	(7.4)

Enhancing the role of family medicine in the health system	159	(77.2)	40	(19.4)	4	(1.9)	3	(1.5)

Note: Not all questions are answered by all respondents

## Discussion

The study showed that the satisfaction of medical professionals was low with offered services and availability and quality of medications and equipments at the health institutions except for availability of sufficient number of health care professionals. Majority of respondents expressed a negative view on the overall health system with the main problems identified in the health system being the weak role of medical research, the weak role of professional associations, the weak role of health education and the low governmental fund allocation for health.

Social insurance of medical care for the poor was identified as the highest priority need for the health system improvement followed by enhancing the role of family medicine in the health system, adopting health insurance system, periodic scientific assessment of medical professionals and better involvement of the MoH and professional associations in controlling the private sector.

This study adds to the limited documented knowledge about the functionality of the regional health system in Iraqi Kurdistan and its priority needs for improvement. It provides an insight to this subject from medical professionals' view through defining the main themes related to its strengths, weaknesses and opportunities for improvement. Given the importance of having the views of medical professionals in any health system reform and the fact that these views are often not looked for, the relevance of this paper might go beyond the specific views of the Iraqi medical professionals that are primarily of local interest and it can serve as a case study which could be followed by others in other contexts.

The study, however, has a number of limitations. The survey targeted only the medical professionals working in Erbil governorate as the limited resources did not allow studying those working in the other two governorates in Kurdistan region; Sulaymaniya and Duhok. Professionals working in other governorates may face different problems and challenges and have different views on the issues included in this survey keeping in mind that the regional MoH is situated in Erbil governorate. Similarly, nurses and other health care workers were not included in this study. These may have different perception and concerns about the health system. Using close-ended questions might have assisted in increasing the response rate due to the simplicity of administration. However, close items do not allow study participants to openly and better express their viewpoints. This limitation was partially addressed through using an initial small scale survey with open-ended questions to develop the close items. Another limitation of the study includes the subjectivity of providers rating the health services while they are the one who deliver such services. The primary focus of the study was the public sector of the health system. Even though the role of private sector in delivering health services in Iraqi Kurdistan is increasingly growing, it was not included in this study. However, we think that this study has partially covered the view from private sector as most Iraqi medical professionals working in the public sector work also in the private sector in afternoon hours.

While the response rate to this survey was satisfactory, the reason why 16.4% failed to respond could be attributed to failure to see the respondents on the next day or follow up visits as many medical professionals have duties in more than one health facility. Medical professionals with stronger views on the need to reform might have more enthusiastically responded to the survey, while those with weaker views might have chosen not to respond.

A number of health system themes derived from the results of this study in relation to problems and priorities for improvement correspond well with those derived from other studies and reports from Iraq. The difficulties and challenges facing the public health facilities in providing quality health services have also been reported by another study [5]. The main problem with human resources in Iraqi health system is not with the number of available staff, but it is related to their uneven distribution and shortage in some specific health professions. An example of this is the excess in specialist physicians and insufficient physicians focusing on the primary health care or family practice [4]. While insufficiency in nurses and other health staff is well documented in the Iraqi health system, the Kurdistan region has the privilege of having better situation in this concern which might be related to having a more respective culture for nurses and thus the nursing job is increasing [11,12]. The inadequate health education activities, which can be attributed to the nonexistence of programs for patient education and possibilities for strengthening self care, has also been identified by a WHO document [3]. The low governmental fund allocation for health agrees with the fact that Iraq's fund allocation for health in 2008 was 4.1% of the gross national product, which accounts for US$87.7 per capita [16]. This makes the country one of the low spending countries on health.

Interestingly a number of additional health system themes in relation to problems and priorities for improvement emerged from this study. The scarcity of medical research and its poor implications in health policy and evidence-based decision making are in fact well recognized problems in most developing countries including Iraq [3,5,17]. The need for social insurance of medical care for the poor is primarily related to the inability of the poor population to afford the cost of the private sector services where most of such services are provided [3]. The need for enhancing the role of family medicine in the health system has emerged as family medicine practice has lately received much attention and has been recognized as a need in many countries, which is attributed to the successful experience in a number of countries particularly in the Middle East and the advocacy of WHO for its adoption [18]. The need for adopting health insurance system is partly related to the increasing role of private sector in providing healthcare services and partly to the large number of marginalized and poor people who can not afford private sector costs [6]. As the public facilities do not provide all health services and due to the load on public facilities many patients need the services of the private sector, which is a problem especially for the poor people due to the high costs of the private sector that need to be out of pocket payment. Since this problem is more related to the poor people, the need for adopting health insurance specifically to poor people was more strongly supported than adoption a general health insurance system.

Periodic scientific assessment of medical professionals is becoming an increasingly recognizable need for health system improvement especially with lack of procedures and guidelines for appraising the staff performance and knowledge in Iraq [5,19]. The requirement for better involvement of the MoH and professional associations in controlling the private sector is again related to the uncontrolled rapid expansion of this sector and its increasing role in health care provision as reported by the WHO [3].

The different problems and priority needs identified in this study can guide and assist policy makers in their efforts to improve the current health system in Iraqi Kurdistan region and in Iraq as whole. The study can also guide researchers to expand on the individual issues recognized in this study and try to better elaborate and understand them.

## Conclusions

The medical professionals had a relatively negative view on different aspects of the health system in Iraqi Kurdistan region, which possibly point out to the challenges the system is facing and the need for major improvements. A number of problems and different priority needs for health system improvement have been recognized that require to be studied in more details.

## Abbreviations

MOH: Ministry of Health; PHC: Primary health care; WHO: World Health Organization;

## Competing interests

The authors declare that they have no competing interests.

## Authors' contributions

TR, SFH and SNP participated in designing the study. TR, SFH carried out the data collection. SNP, AHTS and ATNG carried out the data analysis. SNP and SAM drafted the first version of the paper. AHTS and AT NG extensively reviewed the first draft and made comprehensive changes. All six authors reviewed the final draft and approved it.
